# Avian influenza virus monitoring in wintering waterbirds in Iran, 2003-2007

**DOI:** 10.1186/1743-422X-7-43

**Published:** 2010-02-19

**Authors:** Sasan R Fereidouni, Ortrud Werner, Elke Starick, Martin Beer, Timm C Harder, Mehdi Aghakhan, Hossein Modirrousta, Hamid Amini, Majid Kharrazian Moghaddam, Mohammad H Bozorghmehrifard, Mohammad A Akhavizadegan, Nicolas Gaidet, Scott H Newman, Saliha Hammoumi, Giovanni Cattoli, Anja Globig, Bernd Hoffmann, Mohammad E Sehati, Siamak Masoodi, Tim Dodman, Ward Hagemeijer, Shirin Mousakhani, Thomas C Mettenleiter

**Affiliations:** 1 Friedrich-Loeffler-Institut (FLI), Insel Riems, Germany; 2 Razi Research Institute, Karaj, Iran; 3 Wildlife Bureau, Department of Environment, Tehran, Iran; 4 Clinical Sciences Department, Faculty of Veterinary Medicine, University of Tehran, Tehran, Iran; 5 Centre de Cooperation Internationale en Recherche Agronomique pour le Développement, Montpellier, France; 6 EMPRES Wildlife Unit, Food and Agriculture Organization of the United Nations, Rome, Italy; 7 Research and development Deptartment, Istituto Zooprofilattico Sperimentale delle Venezie, Legnaro, Italy; 8 Biodiversity and Ecological Networks, Wetlands International, Wageningen, the Netherlands; 9 Bird Conservation Society of Iran, Tehran, Iran

## Abstract

**Background:**

Virological, molecular and serological studies were carried out to determine the status of infections with avian influenza viruses (AIV) in different species of wild waterbirds in Iran during 2003-2007. Samples were collected from 1146 birds representing 45 different species with the majority of samples originating from ducks, coots and shorebirds. Samples originated from 6 different provinces representative for the 15 most important wintering sites of migratory waterbirds in Iran.

**Results:**

Overall, AIV were detected in approximately 3.4% of the samples. However, prevalence was higher (up to 8.3%) at selected locations and for certain species. No highly pathogenic avian influenza, including H5N1 was detected. A total of 35 AIVs were detected from cloacal or oropharyngeal swab samples. These positive samples originated mainly from Mallards and Common Teals.

Of 711 serum samples tested for AIV antibodies, 345 (48.5%) were positive by using a nucleoprotein-specific competitive ELISA (NP-C-ELISA). Ducks including Mallard, Common Teal, Common Pochard, Northern Shoveler and Eurasian Wigeon revealed the highest antibody prevalence ranging from 44 to 75%.

**Conclusion:**

Results of these investigations provide important information about the prevalence of LPAIV in wild birds in Iran, especially wetlands around the Caspian Sea which represent an important wintering site for migratory water birds. Mallard and Common Teal exhibited the highest number of positives in virological and serological investigations: 43% and 26% virological positive cases and 24% and 46% serological positive reactions, respectively. These two species may play an important role in the ecology and perpetuation of influenza viruses in this region. In addition, it could be shown that both oropharyngeal and cloacal swab samples contribute to the detection of positive birds, and neither should be neglected.

## Background

Wild waterbirds are considered the main reservoir of all subtypes of avian influenza viruses (AIV). Low pathogenic AIV (LPAIV) are widely distributed in wild avian species around the world. They have been most frequently identified in waterbirds of the orders Anseriformes (including ducks, geese and swans) and Charadriiformes (particularly gulls and terns). These viruses replicate in epithelial cells of the respiratory and intestinal tracts of birds, and are excreted in high concentrations in their faeces [1]. It is now well recognized that global influenza virus surveillance in wild birds is important in understanding the role of wild birds in the epidemiology and ecology of these viruses.

After expansion of HPAIV H5N1 from Southeast Asia into many Eurasian and African countries, the frequency and intensity of avian influenza surveys in the world increased dramatically. In particular North American and European countries gathered massive epidemiological information regarding circulation of AIV in wild birds. Yet, little is known about the prevalence of AIV in wild birds in West & Central Asian countries and the Middle East. Many countries in this region were severely affected by HPAI H5N1 in late 2005 and early 2006, with recurrent outbreaks since 2007 [2]. In Iran, two outbreaks of HPAI H5N1 have been officially reported in wild birds and domestic poultry during 2006 and 2008, respectively.

The wetlands located in the southern part of the Caspian Sea represent major wintering and stopover sites during migration for many wild waterbirds from Siberia and northern Russia. Several million migratory birds usually arrive in October and either remain until February/March or migrate further south.

Here, we describe the results of four years of AIV surveillance in wild birds by using different virological, molecular and serological methods. This study provides the first extensive survey of AIV in wild birds in West and Central Asia and the Middle East.

## Methods

### Sampling plan

**Figure 1:**
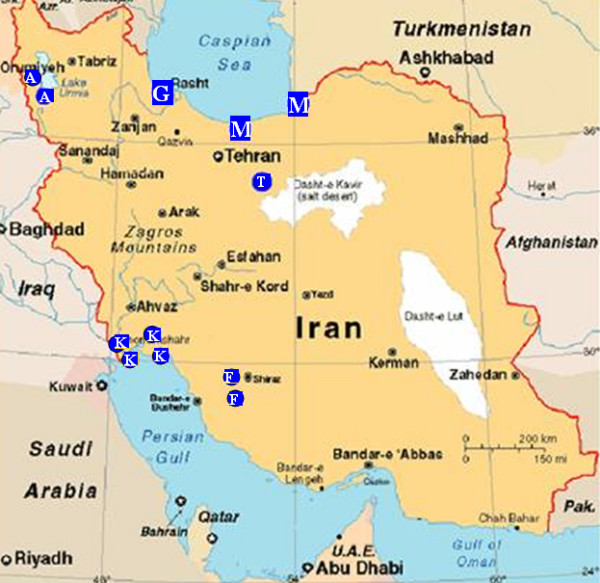
**The geographical distribution of sampling sites in Iran (blue spots; capital letters in the spots indicate the province: A: West Azerbaijan, F: Fars, G: Gilan, K: Khuzestan, M: Mazandaran, T: Tehran)**.

Samples were collected from 1146 waterbirds belonging to 45 species (11 families, Table 1). The samples were mainly obtained from captured or hunted birds, or during ringing activities. Mist nets with mesh sizes of 20 × 20 and 50 × 50 mm were used to capture the birds for sampling. Samples were collected between October and March from 2003 to 2007 at 18 sites located in six provinces of Iran including Mazandaran, Gilan, West Azerbaijan, Tehran, Fars and Khuzestan (Figure 1). The sampling sites comprise the most important wetlands of Iran, serving as wintering sites for migratory waterbirds. The majority of samples (83%) were collected from birds staging in the wetlands along the southern shores of the Caspian Sea which form an important ecological site for wild migratory birds along the Central Asia flyway.

**Table 1:** Wild birds sampled in Iran during different years of study, and AIV positives by rRT-PCR.

Family	Bird name	Scientific name	2003 & 2004	2005	2007	Total Species No.	Species Pos. No.	No. Bird/Family	Pos./Family
Podicipedidae	Black-necked Grebe	*Podiceps nigricollis*	7	-	-	7	0	19	0
			
	Great-crested Grebe	*Podiceps cristatus*	3	1	-	4	0		
			
	Little Grebe	*Podiceps ruficollis*	8	-	-	8	0		

Phalacrocoracidae	Lesser Cormorant	*Phalacrocorax pygmaeus*	-	1	1	2	0	14	0
			
	Great Cormorant	*Phalacrocorax carbo*	11	-	1	12	0		

Ardeidae	Great White Egret	*Egretta alba*	3	2	-	5	0	22	0
			
	Grey Heron	*Ardea cinerea*	6	4	-	10	0		
			
	Little Egret	*Egretta garzetta*	3	4	-	7	0		

Phoenicopteridae	Greater Flamingo	*Phoenicopterus roseus*	8	4	-	12	0	12	0

Anatidae	Gadwall	*Anas strepera*	10	1	15	26	0	745	31
			
	Garganey	*Anas querquedula*	3	-	-	3	1		
			
	Greylag Goose	*Anser anser*	3	-	1	4	1		
			
	Mallard	*Anas platyrhynchos*	78	34	68	180	15		
			
	Northern Pintail	*Anas acuta*	17	1	18	36	1		
			
	Common Pochard	*Aythya ferina*	5	5	8	18	1		
			
	Red-crested Pochard	*Netta rufina*	1	-	-	1	0		
			
	Ruddy Shelduck	*Tadorna ferruginea*	1	5	-	6	0		
			
	Greater Scuap	*Aythya marila*	-	1	-	1	0		
			
	Common Shelduck	*Tadorna tadorna*	10	-	1	11	0		
			
	Northern Shoveler	*Anas clypeata*	10	1	4	15	2		
			
	Common Teal	*Anas crecca*	79	36	243	358	9		
			
	Tufted Duck	*Aythya fuligula*	2	2	1	5	1		
			
	White-headed Duck	*Oxyura leucocephala*	2	-	-	2	0		
			
	Lesser White-fronted Goose	*Anser erythropus*	-	-	1	1	0		
			
	Greater White-fronted Goose	*Anser albifrons*	-	-	1	1	0		
			
	Eurasian Wigeon	*Anas penelope*	4	4	69	77	0		

Rallidae	Common Coot	*Fulica atra*	117	39	77	233	4	234	4
			
	Water Rail	*Rallus aquaticus*	1	-	-	1	0		

Recurvirostridae	Pied Avocet	*Recurvirostra avosetta*	4	-	-	4	0	6	0
			
	Black-winged Stilt	*Himantopus himantopus*	2	-	-	2	0		

Charadriidae	Northern Lapwing	*Vanellus vanellus*	10	1	4	15	0	17	0
			
	White-tailed Lapwing	*Vanellus leucurus*	2	-	-	2	0		

Scolopacidae	Black-tailed Godwit	*Limosa limosa*	3	2	-	5	0	51	0
			
	Dunlin	*Calidris alpina*	5	-	-	5	0		
			
	Jack Snipe	*Lymnocryptes minimus*	3	-	1	4	0		
			
	Marsh Sandpiper	*Tringa stagnatilis*	13	-	-	13	0		
			
	Common Redshank	*Tringa totanus*	12	1	1	14	0		
			
	Common Greenshank	*Tringa nebularia*	-	5	-	5	0		
			
	Kentish Plover	*Charadrius alexandrinus*	-	2	-	2	0		
			
	Ruff	*Philomachus pugnax*	1	2	-	3	0		

Laridae	Black-headed Gull	*Larus ridibundus*	16	1	-	17	0	25	0
			
	Little Gull	*Larus minutus*	3	-	-	3	0		
			
	Slender-billed Gull	*Larus genei*	2	-	-	2	0		
			
	Yellow-legged Gull	*Larus cachinnans*	3	-	-	3	0		

Sternidae	Whiskered Tern	*Chlidonias hybridus*	1	-	-	1	0	1	0

			**472**	**159**	**515**	**1146**	**35**	**1146**	**35**

During 2003-2005 only cloacal samples (n = 631) and in 2007 cloacal and oropharyngeal samples were collected. In addition, 711 serum samples were collected from 27 different species (Tables 2 &3). In 2006, sampling was not permitted due to an HPAI H5N1 outbreak in the wild bird population in Iran.

**Table 2:** Time and location of sampling and prevalence of virological and serological AIV positive wild birds during 2003-2007.

Year	Month	Province	Swab Samples	Pos.	Serum Samples	Pos.
2003	Feb.	Khuzestan (K)	30	1	0	0
	
	Nov.	W. Azerbaijan (A)	58	0	4	1
	
	Nov.	Tehran (T)	12	1	0	0
	
	Nov.	Gilan (G)	118	3	82	22
	
	Dec.	Mazandaran (M)	49	0	40	23
	
	Dec.	Gilan	52	0	47	20
	
	Dec.	Fars (F)	63	0	36	8

2004	Jan.	Tehran	13	0	0	0
	
	Feb.	Khuzestan	26	0	5	2
	
	Feb.	Tehran	51	7	3	1

2005	Jan	Mazandaran	22	0	0	0
	
	Feb.	Tehran	59	1	11	5
	
	Mar.	Mazandaran	78	1	32	4

2007	Feb.	Gilan	27	0	23	7
	
	Feb.	Mazandaran	488	21	428	252

			**1146**	**35**	**711**	**345**

**Table 3:** AIV serological results of different bird species at different sampling times.

Family	Bird species	2003/2004		2005		2007		Total		Number/family	
		
		Tested	Pos.	Tested	Pos.	Tested	Pos.	Tested	Pos.	Tested	Pos.
Podicipedidae	Black-necked Grebe	1	0	-	-	-	-	1	0	8	0
			
	Great-crested Grebe	3	0	-	-	-	-	3	0		
			
	Little Grebe	4	0	-	-	-	-	4	0		

Phalacrocoracidae	Great Cormorant	7	1	-	-	-	-	7	1	7	1

Ardeidae	Great White Egret	2	1	2	0	-	-	4	1	14	2
			
	Little Egret	2	1	1	0	-	-	3	1		
			
	Grey Heron	4	0	3	0	-	-	7	0		

Phoenicopteridae	Greater Flamingo	3	3	2	1	-	-	5	4	5	4

Anatidae	Gadwall	1	0			13	4	14	4	521	313
			
	Greylag Goose	2	2	-	-	-	-	2	2		
			
	Mallard	32	28	10	3	66	50	108	81		
			
	Northern Pintail	11	5	1	0	17	16	29	21		
			
	Common Pochard	4	1	1	0	6	6	11	7		
			
	Ruddy Shelduck	-	-	3	2	-	-	3	2		
			
	Northern Shoveler	6	5	-	-	4	3	10	8		
			
	Common Teal	41	22	6	2	225	133	272	157		
			
	Eurasian Wigeon	2	0	2	0	68	31	72	31		

Rallidae	Common Coot	66	1	11	0	49	16	126	17	126	17

Recurvirostridae	Pied Avocet	1	1	-	-	-	-	1	1	1	1

Charadriidae	Northern Lapwing	5	0	-	-	3	0	8	0	8	0

Scolopacidae	Black-tailed Godwit	2	0	-	-	-	-	2	0	7	3
			
	Common Redshank	4	2	-	-	-	-	4	2		
			
	Common Greenshank	-	-	1	1	-	-	1	1		

Laridae	Black-headed Gull	10	4	-	-	-	-	10	4	14	4
			
	Little Gull	1	0	-	-	-	-	1	0		
			
	Slender-billed Gull	1	0	-	-	-	-	1	0		
			
	Yellow-legged Gull	2	0	-	-	-	-	2	0		

		**217**	**77**	**43**	**9**	**451**	**259**	**711**	**345**	**711**	**345**

In 2007, in the framework of an international collaboration, birds were sampled in duplicate and tested independently by reference laboratories of the World Organisation for Animal Health (OIE) at the Friedrich-Loeffler-Institut (FLI), Germany and the Istituto Zooprofilattico Sperimentale delle Venezie (ISZ-Ve), Italy, as well as at the Agricultural Research Centre for International Development (CIRAD), France. Cloacal and oropharyngeal samples were collected with cotton swabs, stored in viral transport medium (Hank's medium or PBS) containing antibiotics and antimycotics (plus 5% calf serum during 2003-2005) and maintained at -70°C after arrival at the laboratory. Serum samples were stored at -20°C until tested.

### Diagnostic procedures

The samples from 2003-2004 were analyzed by virus isolation (VI) at the Razi Institute, Iran, and real-time reverse transcription PCR (rRT-PCR) at the Friedrich-Loeffler-Institut (FLI), Germany, whilst positive samples were further characterized at the FLI. The samples from 2005 were analysed only by virus isolation (at the Razi Institute) and two positive samples were further characterized at the FLI.

In 2007, rRT-PCR was performed for screening and only PCR-positive samples were processed for virus isolation. Samples duplicated in the field were analysed at the FLI (rRT-PCR and VI) and at CIRAD (France) (rRT-PCR) and the IZS-Ve (Italy) (VI).

Isolates were characterized by conventional hemagglutination inhibition (HI) and neuraminidase inhibition (NI) assays, and subsequently confirmed by subtype specific RT-PCR assays and sequencing. The subtypes of PCR-positive but isolation-negative samples, were determined by subtype specific RT-PCR, DNA microarray and sequencing.

### Virus isolation and characterization

Virus isolation was carried out in specific pathogen free (SPF) embryonated chicken eggs based on standard procedures [3].

### RNA extraction, RT-PCR and Real-time RT-PCR

RNA was extracted either using the QIAamp Viral RNA kit (Qiagen) for swab materials (field samples), or the High Pure Viral RNA kit (Roche) for virus isolates (allantoic fluids), according to the manufacturer's instructions. The 2007 samples were processed by automated RNA extraction (Freedom Evo 3000, Tecan) using the NucleoSpin 96 Virus Core kit (Macherey & Nagel).

Reverse transcription-PCR (RT-PCR) assays were performed on the basis of one-step protocols using appropriate RT-PCR Kits (Qiagen or Invitrogen) according to the manufacturers' instructions. Subtype specific RT-PCR assays using specific primers for different HA [4] and NA [5] were used for subtype identification or confirmation. Degenerate consensus primers were used for full length amplification and further sequencing of different viral segments [6].

The samples were tested by a modified TaqMan one-step real-time RT-PCR assay targeting the influenza A virus M gene [7], an H5 subtype gene fragment and an H7 subtype gene fragment [3]. Brilliant QRT-PCR kit (Stratagene), SuperScript III One-step RT-PCR kit with Platinum Taq DNA polymerase (Invitrogen) and one-step RT-PCR kit (Qiagen) were used on a MX3000P Real-Time PCR System (Stratagene). In all tests, negative RNA preparation controls and negative and positive rRT-PCR controls as well as an internal transcription and amplification control (IC-2) were included [8].

### Sequencing and phylogenetic analyses

PCR products of the anticipated size range were purified from agarose gels using the QIAquick Gel Extraction Kit (Qiagen). Purified DNA fragments were cycle-sequenced in both directions using the same primers as for RT-PCR. The Prism Big Dye Terminator v1.1 cycle sequencing kit (Applied Biosystems) was used and amplicons were analysed on an automatic sequencer (ABI-377, Applied Biosystems). Assembled nucleotide sequences were then used in BlastN2 database searches for subtype specification. Phylogenetic analyses were carried out for complete open reading frame of HA gene of selected H9N2 AIV using the neighbour-joining (NJ) method, with 1000 bootstrap replicates implemented in the MEGA 4 programme [9].

### Hemagglutination (HA) and Neuraminidase inhibition (NI) assay

HA assay was performed based on standard protocols [3] using reference antisera prepared from 30 different viruses representing all 16 avian influenza HA subtypes and 9 different avian paramyxoviruses. The previously described NI assay was used for determination of the NA subtype of virus isolates from 2003-2004 [5].

### Pathogenicity assessment

Two isolates from 2003 and 2004 of subtypes H7N3 and H9N2 were selected for IVPI testing [3] due to the potential pathogenicity of these subtypes for domestic poultry. Pathogenicity of two H5 isolates from 2007 was determined by sequencing of the HA cleavage site and restriction enzyme cleavage pattern (RECP) assay [10].

### Competitive ELISA

An in-house competitive ELISA method was used for testing of 217 serum samples from 2003-2004 [11]. For the investigation of 494 serum samples collected during 2005-2007 (Table 4), a cELISA kit based on the same assay principle was used (ID Screen, Influenza A NP Antibody Competition, ID.VET). The cut-off value of this test was used as recommended by the suppliers.

**Table 4:** AIV characterized from different species of wild birds during 2003-2007 in Iran.

Year/Month	ID-code	Sample type	subtype		Bird species	Virus isolation	Province
2003/Feb	K8	C	H9	N2	Garganey	Yes	Khuzestan

2003/Nov	T9	C	?	N7	Common Coot		Tehran

2003/Nov	G52	C	?	?	Northern Shoveler		Gilan

2003/Nov	G54	C	H9	N2	Mallard	Yes	Gilan

2003/Nov	G94	C	H9	N2	Northern Shoveler	Yes	Gilan

2004/Feb	V4	C	?	?	Common Teal		Tehran

2004/Feb	V10	C	H3	N8	Mallard	Yes	Tehran

2004/Feb	V15	C	H10	N7	Mallard	Yes	Tehran

2004/Feb	V16	C	H8	N4	Mallard	Yes	Tehran

2004/Feb	V17	C	?	?	Mallard		Tehran

2004/Feb	V31	C	H7	N3	Mallard	Yes	Tehran

2004/Feb	V40	C	H10	N7	Mallard	Yes	Tehran

2005/Feb	V41	C	H10	N7	Mallard	Yes	Tehran

2005/Mar	M72	C	H7	N7	Mallard	Yes	Mazandaran

2007/Feb	T31	T	?	?	Common Coot		Mazandaran

2007/Feb	T41	T	?	?	Mallard		Mazandaran

2007/Feb	C54	C	H8	N4	Greylag Goose		Mazandaran

2007/Feb	C64	C	H1	N1	Common Teal		Mazandaran

2007/Feb	C108	C	H5 H11	N3 N9	Common Teal		Mazandaran

2007/Feb	C136	C	H1	N1	Common Teal	Yes	Mazandaran

2007/Feb	T149	T	H6	N2	Common Teal		Mazandaran

2007/Feb	C154	C	H11	N1	Common Teal		Mazandaran

2007/Feb	T183	T	H11	?	Common Teal		Mazandaran

2007/Feb	T223	T	H10	N8	Common coot		Mazandaran

2007/Feb	T292	T	H5	?	Common Pochard		Mazandaran

2007/Feb	C303	C	H3	?	Tufted Duck		Mazandaran

2007/Feb	C309	C	H10	N8	Common Coot		Mazandaran

2007/Feb	C364	C	H9	N2	Mallard		Mazandaran

2007/Feb	T366	T	H9	N2	Mallard		Mazandaran

2007/Feb	T367	T	H9	N2	Mallard		Mazandaran

2007/Feb	T370/C370	C & T	H9	N2	Mallard	Yes	Mazandaran

2007/Feb	T371	T	H9	N2	Mallard		Mazandaran

2007/Feb	C381	C	H11	N9	Northern Pintail		Mazandaran

2007/Feb	C388	C	H3	?	Common Teal		Mazandaran

2007/Feb	C415	C	H11	N2	Common Teal		Mazandaran

C: cloacal. T: oropharyngeal

### Microarray

Microarray was used for HA and NA subtyping of samples with low viral load and which therefore did not yield enough PCR amplificates for PCR subtyping or sequencing. Sample RNA was amplified by RT-PCR assays using biotinylated generic pan HA and pan NA primers. An *in vitro *transcript 'LPC-pan HA' and a 'no template control' were included in every run. Microarray detection of the biotinylated PCR products was done as described by Gall *et al. *[12].

### Western blot analysis

Western blot analysis was carried out according to Kothlow *et al. *[13], to support the cELISA results of selected serum samples. Briefly, after separation of purified virus by sodium dodecyl sulphate-polyacrylamide gel electrophoresis proteins were blotted onto nitrocellulose membrane, and strips were incubated with 1:250 diluted test and control sera overnight. After the respective washing steps, bound duck immunoglobulin (Ig) was detected by mAb (1:2000 dilution) recognising Ig of several species followed by incubation with horseradish peroxidase-conjugated goat-anti-mouse Ig-specific polyclonal antibody (Sigma). Finally, strips were allowed to react with a horseradish peroxidase substrate (ECL plus Western Blotting Detection System, Amersham Biosciences) and the reaction was visualized by autoradiography on X-ray film. Sera which reacted at least with either the NP or the M protein were considered positive. Sera from 8 mallards hatched and kept under quarantine, were used as Western blot negative controls.

## Results

### Detection of LPAIV in Iran

In total 3% of all sampled birds were AIV positive by rRT-PCR of M gene (Table 1). Out of 11 bird families examined, two were positive, Anatidae and Rallidae (Tables 1 &2). The highest number of AIV (31 out of 35 rRT-PCR positive samples) were detected in dabbling ducks (genus Anas) including Mallard *Anas platyrhynchos *(n = 15; 8.3%), Common Teal *Anas crecca *(n = 9; 2.5%) and Northern Shoveler *Anas clypeata *(n = 2). Individual Greylag Goose *Anser anser*, Garganey *Anas querquedula*, Northern Pintail *Anas acuta*, Common Pochard *Aythya ferina *and Tufted Duck *Aythya fuligula *also yielded positive results. Out of 45 sampled bird species, eight were positive. In total, Anatidae made up 65% of the sample volume, and 4.3% of them were AIV positive. Four Common Coots *Fulica atra *of the Rallidae family were also AIV positive in this study (1.7%).

### Temporal and geographical distribution of AIV

**Figure 2:**
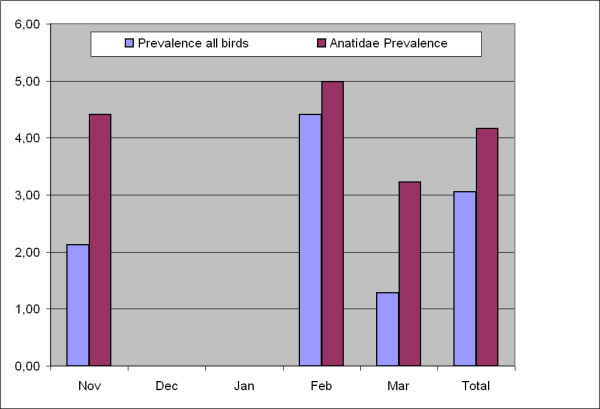
**Prevalence (%) of AIV in total sampled birds and in Anatidae in different months of sampling in Iran**.

During five months of sampling in different years and different provinces, the highest numbers of positive samples were found in February and November (Figure 2). The number of samples collected in this study was not distributed evenly over different months. Approximately 66% of samples (n = 759) were collected during February and early March (2004, 2005 and 2007). 88% of positive samples were found in this portion of samples, though 78% of samples collected during this period originated from ducks.

Out of the 35 AIV-positive samples detected during the four year study, 34.3% (n = 12) were found in 2003/2004, 5.7% (n = 2) in 2005 and 60% (n = 21) in 2007. 84% of samples in 2007 belonged to Anatidae (compared to 57% in 2005 and 48% during 2003/2004); and only in 2007 both oropharyngeal and cloacal samples were collected from each bird. Prevalence of AIV was 2.54% in 2003/2004, 1.26% in 2005 and 4.08% in 2007.

Out of 21 AIV-positive samples in 2007 (the year in which paired sampling from 515 birds was carried out), nine samples were positive only in the oropharyngeal swab, eleven samples positive only in the cloacal swab, and only one bird positive in both oropharyngeal and cloacal swab samples. The highest number of positive samples originated from the wetlands south of the Caspian Sea (n = 25) and in a seasonal wetland in the south-east of Tehran province (n = 9).

### Detection of specific AIV antibodies

The results of serological investigation of 711 serum samples are shown in Tables 2 and 3. The seroprevalence rates against AIV were 35.5%, 21% and 57.4% respectively for 2003/2004, 2005 and 2007. However, the composition of bird species in these four sampling periods was different. During 2003-2005 only 45.6% of serum samples belonged to Anatidae, which increased to 88.5% in 2007. However, the proportion of positive Anatidae in 2004/2005 and 2007 samples was almost similar (64% and 61% respectively). Anatidae contributed a high proportion of positive results: 81 out of 108 Mallard, 157 out of 272 Common Teal, 8 out of 10 Northern Shoveler, 7 out of 10 Common Pochard, 21 out of 28 Northern Pintail, 31 out of 70 Eurasian Wigeon and 2 out of 2 Greylag Goose were antibody-positive. In total, birds from 17 of 25 species carried antibodies against AIV (Table 3). A randomly selected batch of 31 cELISA positive and negative serum samples belonging to different species was tested by Western blotting with a high correlation between the two tests (data not shown).

### Virus isolation and characterisation

In total, 35 LPAIV were molecularly identified from 1601 oropharyngeal and cloacal samples originating from 1146 different wild waterbirds wintering/staging in Iran. No highly pathogenic strains, including H5N1, were detected in this survey. A total of twelve AIV were isolated and subtyped mainly during the 2003/2004 investigation; virus isolation for the additional 23 molecularly positive samples (mainly during the 2007 investigation) failed even after 2-3 passages in SPF chicken eggs (Table 3). HA and/or NA subtypes of 17 samples from this group were characterized by subtype-specific RT-PCR, sequencing and microarray (Table 4).

**Figure 3:**
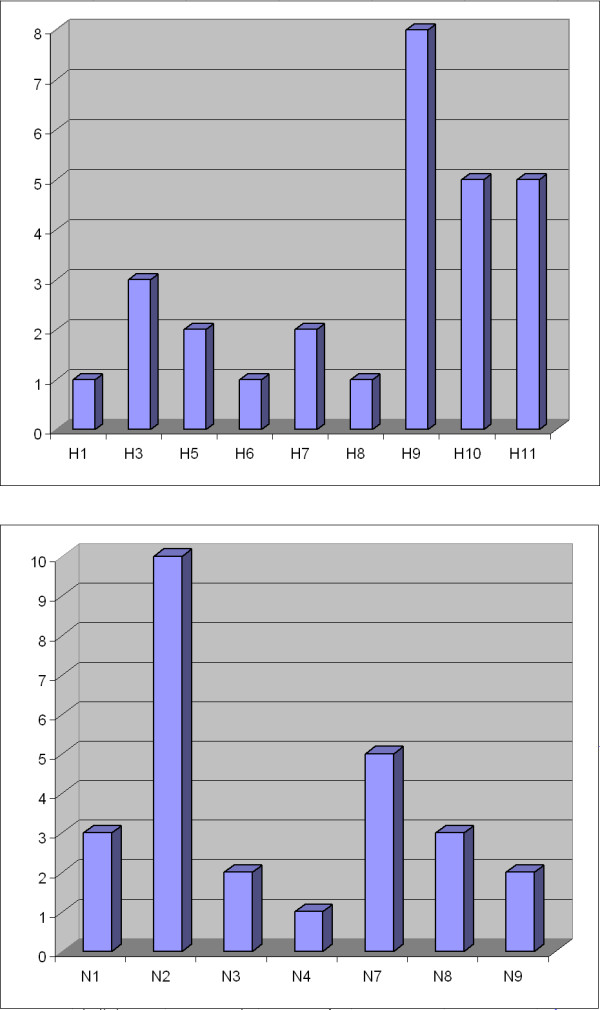
**Frequency of different hemagglutinin and neuraminidase subtypes identified in wild bird samples during 2003-2007**.

Characterized AIV were categorised into 14 subtypes using HI, NI, subtype-specific RT-PCR, microarray and sequencing: H1N1, H3N8, H5N9, H6N2, H7N3, H7N7, H8N4, H9N2, H10N7, H10N8, H11N1, H11N2, H11N3, and H11N9. The most common HA subtypes were H9, H10 and H11, and the most common NA subtypes were N2, N7 and N1 (Figure 3). Two LPAIV H5 and two LPAIV H7 strains were identified. One cloacal sample taken from a Common Teal was positive for two different AIV subtypes: H5N3 (or N9) and H11N9 (or N3).

One H7N3 and one H9N2 isolate (from 2003/2004) were assessed as LPAIV using IVPI test, as no evidence of disease or pathogenicity in SPF chickens was observed (IVPI = 0). Two H5 subtype viruses, from 2007, were considered as LPAIV in RECP assay. Sequencing of the HA cleavage site for the H5 and H7 viruses confirmed their status as LPAI viruses. The amino acid patterns of the HA cleavage site for H5 and H7 subtype viruses were RETR*G and PKGR*G, respectively.

### Inter-laboratory real-time PCR results

All 515 samples from 2007 were tested independently by two AI reference laboratories (FLI tested one set of samples, ISZ-Ve and CIRAD together another set) using real-time RT-PCR (Tables 1 &3). Results for most of the samples with moderate and strongly positive results (Ct-values ≤ 33) were identical, and only 4 weakly positive samples (33.3 ≤ Ct-values ≤ 35.5) were found positive by only one laboratory. Positive and negative controls included in all tests ensured the validity of rRT-PCR results. We showed that double sampling from the wild waterbirds is possible and the rRT-PCR results revealed a very high degree of agreement.

### Phylogenetic analyses

**Figure 4:**
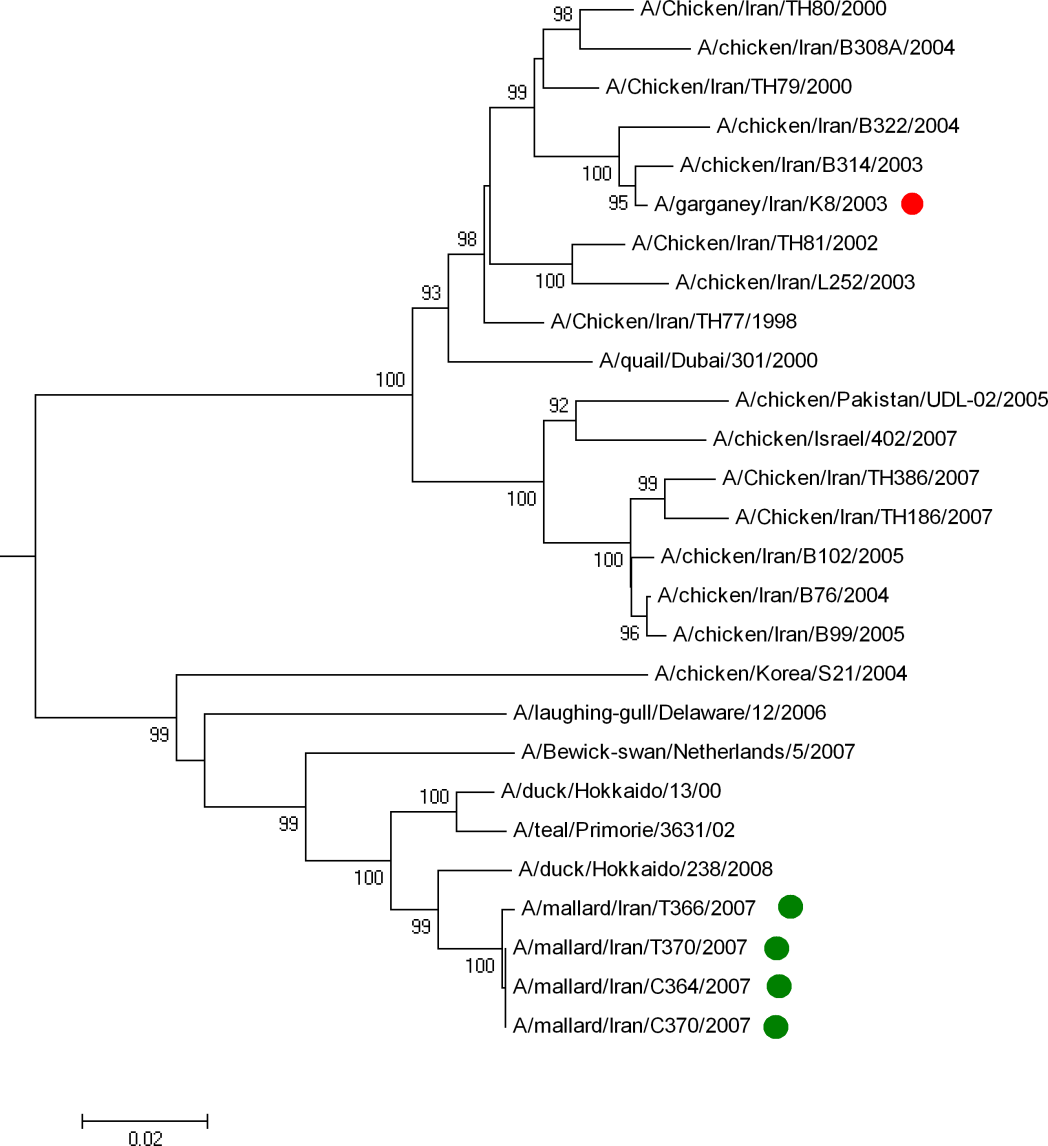
**Phylogenetic tree for the full length hemagglutinin gene of H9N2 influenza viruses**. The tree constructed by Neighbour-joining method. Sequences obtained in this study were labelled by red (2003 virus) and green dots (2007 viruses). The other sequences were selected from GenBank. The numbers represent bootstrap values which were determined using 1000 replications.

In total, nine H9N2 AIV were identified in samples collected during our monitoring studies, and the HA genes of five of them were sequenced (GenBank: FN600116 - FN600119). Due to widespread infection of poultry farms in Iran with H9N2 subtype virus, phylogenetic analysis was preferentially carried out on these viruses. A/Garganey/Iran/G8/2003 (H9N2) was isolated from a Garganey in the southern part of Iran which was suspected to have been kept together with backyard poultry for a short time before sampling. The four 2007 H9N2 viruses were identified in a small group of Mallards in the northern part of Iran, without any known contact with domestic poultry. The 2003 virus clustered closely with H9N2 viruses isolated in poultry in Iran during 1999-2003, while the 2007 viruses clustered together with wild bird H9N2 viruses from Russia and Hokkaido (Figure 4).

## Discussion

The important role of waterbirds, especially waterfowl, as a reservoir for avian influenza viruses of all subtypes is well known from intensive investigations from many regions of the world [14-17]. Avian influenza monitoring of wild birds in natural habitats and in areas at risk of transmission between domestic poultry and wild birds will increase the knowledge of epidemiology, ecology and genetic relationships of AIV infections. This knowledge will facilitate risk assessments concerning poultry and wild bird populations and provides information on currently circulating AIV which might also have the potential to become important for human health. However, little information is available about the circulation of influenza viruses in waterbirds in West and Central Asia and in the Middle East. This is the first study of AIV-investigations in wild birds in Iran and this geographic region.

In a survey during 1999-2000 in Northern Europe, 2.6% of wild ducks and 1.4% of wild geese were positive in rRT-PCR [18]. In more recent monitoring studies of wild birds during 2003-2005 in Italy, 5.1% of Anseriformes were positive in rRT-PCR [19]. Also, in an AIV screening in 2005 in Norway, 13.2% of Anseriformes were positive in rRT-PCR [20]. The prevalence of LPAIV in wild birds in Alaska and Canada seems to be more variable [21-23]. The results from these studies have shown that the prevalence of AIV in wild birds, and especially ducks, depends on various factors, including geographic altitude of sampling area, bird species, seasonal parameters and different sample processing approach. In our investigation, the number of positive birds varied based on species, sampling month and sites. In total 3.4% of all sampled birds were positive, but different families and species had different number of positives. Only two out of 11 investigated families (Anatidae and Rallidae) were AIV positive, and among 17 sampled species of Anatidae, 8 species revealed positive results. Prevalence rates for Mallard, Common Teal and Common Coot, with high sample sizes, were 8.3%, 2.5% and 1.7% respectively (Table 1). No positive samples were found in shorebirds (Recurvirostridae, Charadriidae & Scolpacidae).

Previous studies revealed high virus prevalence during the autumn season in the Northern Hemisphere [16], whereas the lowest prevalence rates have been measured in early spring. In contrast, 88% of positive samples in our study were found during February and early March. Interestingly, 78% of samples which were collected in this period came from Anatidae.

The geographical distribution of positive samples reveals further significant differences. In Mazandaran (one of the northern provinces), 21 out of 637 samples tested positive (3.3%), while in a small wetland in the southeast of Tehran province, 9 out of 135 sampled birds were positive (6.7%, Table 2). Sample numbers per species largely reflect the proportion of the wintering populations in different geographical regions of Iran. However, with respect to statistical inferences, a bias regarding different species, seasons and geographical regions cannot be excluded.

The results of this study regarding the dominant AIV infected wild bird species are consistent with several other investigations from Europe and the Americas. Dabbling ducks were found infected with LPAIV at higher prevalence rates than other taxonomic groups [23-25]. Similarly, the highest number of positive AIV samples has been reported from Mallard and Common Teal, relative to any other species [20,23]. Mallards are the most frequently sampled species in most wild birds surveillance studies in the northern hemisphere, though in our study Common Teal and Common Coot occupied a higher ranking (Table 1).

No evidence was found for HPAIV H5N1 circulating in wild birds before (2003-2005) and after introduction of this virus into wild bird and poultry populations in countries around the Caspian Sea in 2006. Nevertheless, the small sample sizes of the species investigated here in comparison to their total populations make it very hard to exclude a low or very low prevalence of HPAIV in wild bird populations. The largest sample sizes in our study belonged to Common Teal, Common Coot and Mallard with 358, 233 and 180 birds, respectively.

The results of recent wild bird surveillance in Sweden indicate that the proportion of positive cloacal samples exceeds positive oropharyngeal samples [26]. Our results show a similar proportion of positive oropharyngeal and cloacal samples. Among 21 positive samples from 2007, nine were only positive in oropharyngeal swabs, 11 were only positive in cloacal swabs, and one was positive in both (Table 3). Only in February 2007 oropharyngeal and cloacal samples were collected, when nearly half of the positive samples were retrieved from oropharyngeal swab samples. In previous years only cloacal swabs had been taken. This may indicate that the number of LPAIV positive birds may be significantly underestimated when relying exclusively on cloacal samples. In addition, it cannot be excluded that different AIV subtypes may exhibit different tissue tropism. For example, out of five H11 subtype viruses detected during 2007, four originated from cloacal samples.

The real-time RT-PCR results of inter-laboratory analyses of 2007 samples were highly concordant, when two identical sets of the samples were tested by laboratories in Germany and France/Italy. This finding shows the robustness of results when using the standard protocols in different laboratories. In addition, it shows sampling of cloaca or oropharyngeal area of the same bird twice, does not cause false negative results of the second sample.

The potentially higher sensitivity of rRT-PCR compared to virus isolation has been mentioned before in other investigations [18]. Although some investigators could demonstrate that avian viruses originating from wild bird samples require several passages in embryonated eggs for adaptation, our investigation during 2003-2005 showed acceptable results even in the first egg passage (Table 3). During the 2007 investigation, only two out of 21 positive samples were also positive in virus isolation. The efficacy of virus isolation was dramatically reduced compared to 2003-2005. This might be due to many factors including: improper sample transfer in 2007 with extended international transport and interruption of the cold chain, quality and preservation of VTM and viral load of the samples.

Validation of a serological assay for detection of AIV-specific antibodies in wild birds is still a matter of debate [15,18,27-32]. Only limited information is available about the sensitivity of the HI assay in these species. In a recent study [33], only 16.9% of wild duck sera which tested positive in a double antibody sandwich blocking ELISA were positive in HI assay. Previous studies showed that competitive ELISAs on the basis of recombinant AIV-NP antigen appear to be a valid and reliable method for testing different bird species for avian influenza infection [15,32,34].

In this study, the overall sero-prevalence based on 711 serum samples from different species was 48.5%, and Anseriformes provided 61.45% positive c-ELISA results. Therefore, the overall sero-prevalence could be misleading due to the species composition of samples. Two duck species with high numbers of samples were Mallards with 75% positive of 108, and Common Teal with 57.7% positive of 272 tested birds. These results, together with the virological data, again emphasize the importance of these species for the epidemiology of AIV in the nature, but the high percentage of AIV positive Mallard and Common Teal may just reflect an over-representation of these species in the sample composition due to their large wintering population in Iran. Thus, although the sample size for other members of this order was small, the detected prevalence rates are impressive (Table 3). Interestingly, only one serum sample from 66 Common Coots sampled during 2003-2005 was weakly seropositive, whereas sixteen from 49 Common Coot samples collected in 2007 were seropositive (32%). In addition, in 2003-2005 we could not find any AIV positive samples from 117 Common Coots taken from six different provinces [35], whereas in 2007 three out of 77 samples from Common Coots were positive in rRT-PCR. The relevance of these findings is not clear. Although seropositive samples were found among only 12% of Non-Anseriformes in 5 different families, some species, such as Greater Flamingo (3/3 positive) and Black-headed Gull (4/10 positive), demonstrated high prevalence rates.

## Conclusions

In summary, the results of these investigations provide important information about the prevalence of LPAIV in wild birds in Iran, especially wetlands around the Caspian Sea which represent an important wintering site for migratory water birds. In addition, it could be shown that both oropharyngeal and cloacal swab samples contribute to the detection of positive birds, and neither should be neglected. Proper sample handling and maintenance of cold chains are important to ensure sample quality which is essential at least for virus isolation. Results obtained by rRT-PCR are less dependent on sample quality as shown by higher number of positives and by the high degree of correlation of results from different laboratories. The obvious problems with virus isolation highlight the ongoing demand of molecular methods for the sensitive detection and characterization of AIV.

## Abbreviations

AIV: avian influenza virus; cELISA: competitive enzyme linked immunosorbent assay; HA: hemagglutinin; HI: hemagglutination inhibition; HPAIV: highly pathogenic avian influenza virus; Ig: immunoglobulin; IVPI: intravenous pathogenicity index; LPAIV: low pathogenic avian influenza virus; mAb: monoclonal antibody; NP-cELISA: nucleoprotein-specific competitive ELISA; NA: neuraminidase; NI: neuraminidase inhibition; NJ: neighbour-joining; PBS: phosphate buffered saline; RECP: restriction enzyme cleavage pattern; rRT-PCR: real time reverse-transcription polymerase chain reaction; RT-PCR: reverse-transcription polymerase chain reaction; SPF: specific pathogen free; VI: virus isolation.

## Competing interests

The authors declare that they have no competing interests.

## Authors' contributions

OW, ES, TCH, HM, NG, SH, GC, AG, BH and SRF contributed to laboratory analyses (serological investigation, virus isolation, molecular diagnostic and sequencing). HA, HM, MKM, SM, MES and SM contributed to field work, sample collection and ornithological data. SHN, WH, HA, MKM, MA, MB and TCM contributed to project management. MA, MAA and MMB contributed to experimental design and statistical analysis.  TCM, SHN, ES, TD, MB, TCH and NG contributed to writing and editing of the manuscript.  SRF was the overall project coordinator and contributed to experimental design, sampling, data analysis and drafting the manuscript.  All authors read and approved the final manuscript.
